# Efficacy of dapagliflozin versus sitagliptin on cardiometabolic risk factors in Japanese patients with type 2 diabetes: a prospective, randomized study (DIVERSITY-CVR)

**DOI:** 10.1186/s12933-019-0977-z

**Published:** 2020-01-07

**Authors:** Ayako Fuchigami, Fumika Shigiyama, Toru Kitazawa, Yosuke Okada, Takamasa Ichijo, Mariko Higa, Toru Hiyoshi, Ikuo Inoue, Kaoru Iso, Hidenori Yoshii, Takahisa Hirose, Naoki Kumashiro

**Affiliations:** 1 Division of Diabetes, Metabolism, and Endocrinology, Department of Medicine, Toho University Graduate School of Medicine, 6-11-1 Omori-Nishi, Ota-ku, Tokyo, 143-8541 Japan; 2 Division of Diabetes, Endocrinology and Metabolism, Tokyo Metropolitan Cancer and Infectious Diseases Center Komagome Hospital, Tokyo, Japan; 3 The First Department of Internal Medicine, School of Medicine, University of Occupational and Environmental Health, Kitakyushu, Japan; 4 Department of Diabetes and Endocrinology, Saiseikai Yokohamashi Tobu Hospital, Kanagawa, Japan; 5 Division of Diabetes and Endocrinology, Japanese Red Cross Medical Center, Tokyo, Japan; 6 Department of Endocrinology and Diabetes, School of Medicine, Saitama Medical University, Saitama, Japan; 7 Department of Internal Medicine, Japan Community Health Care Organization Tokyo Kamata Medical Center, Tokyo, Japan; 8 Department of Medicine, Diabetology and Endocrinology, Juntendo Tokyo Koto Geriatric Medical Center, Tokyo, Japan

**Keywords:** Type 2 diabetes, Cardiometabolic risk factors, Dapagliflozin, Sitagliptin, Glycemic control, Weight loss, Hypoglycemia

## Abstract

**Background:**

Few prospective studies have compared the cardiovascular benefits of sodium-glucose cotransporter-2 (SGLT2) inhibitors and dipeptidyl peptidase 4 (DPP-4) inhibitors. We aimed to clarify the efficacy of dapagliflozin versus sitagliptin for modulating cardiometabolic risk factors including high glycated hemoglobin (HbA1c) levels, hypoglycemia, and body weight.

**Methods:**

This prospective, randomized, open-label, blinded-endpoint, parallel-group trial enrolled 340 Japanese patients with early-stage type 2 diabetes receiving metformin alone or no glucose-lowering agents, who were randomized to receive dapagliflozin or sitagliptin for 24 weeks. The primary endpoint was the proportion of patients who achieved the composite endpoint of HbA1c level maintenance < 7.0% (53 mmol/mol), avoidance of hypoglycemia (maintenance of sensor glucose ≥ 3.0 mmol/L or ≥ 54 mg/dL), and ≥ 3.0% body weight loss from baseline. Secondary endpoints included components of the primary endpoint, other metabolic indices, and glucose variability indices measured using flash glucose monitoring.

**Results:**

Clinical characteristics of patients were age, 58.1 ± 12.2 years; known duration of diabetes, 5.8 ± 6.1 years; body weight, 74.7 ± 14.2 kg; body mass index, 27.9 ± 4.1 kg/m^2^; and HbA1c level, 7.8 ± 0.8% at baseline. The achievement ratio of primary endpoint was significantly higher in the dapagliflozin group than in the sitagliptin group (24.4% vs. 13.8%, P < 0.05). While the rates of HbA1c level maintenance < 7.0% (53 mmol/mol) and avoidance of hypoglycemia were comparable between the groups (49.4 vs. 50.0% and 88.7 vs. 92.3% for dapagliflozin vs. sitagliptin, respectively), body weight loss of ≥ 3.0% was significantly achieved in the dapagliflozin group (54.4 vs. 19.6%, *P *< 0.001). Moreover, dapagliflozin was superior to sitagliptin regarding several secondary endpoints that modulate cardiometabolic risk, namely reducing fasting plasma glucose, insulin, uric acid, increasing high-density lipoprotein cholesterol, and suppressing the increase in serum creatinine and the decrease in estimated glomerular filtration rate. On the other hand, sitagliptin was superior to dapagliflozin in suppressing glucose variability.

**Conclusions:**

Compared to sitagliptin, dapagliflozin was significantly more effective at improving cardiometabolic risk factors, suggesting that SGLT2 inhibitors might be more suitable than DPP-4 inhibitors for preventing cardiovascular events in patients with early-stage but inadequately controlled type 2 diabetes.

*Trial registration* Trial number, UMIN000028014; registered on June 30, 2017

## Background

Approximately 415 million people worldwide are currently living with diabetes, and the prevalence of type 2 diabetes is increasing [1]. Type 2 diabetes is associated with micro- and macrovascular complications leading to cardiovascular diseases [2, 3], which increase the mortality in this population [3]. Thus, the management of patients with type 2 diabetes should focus not only on lowering blood glucose levels but also on preventing vascular complications.

Among the various medications available for type 2 diabetes, oral glucose-lowering agents such as dipeptidyl peptidase 4 (DPP-4) inhibitors and sodium-glucose cotransporter-2 (SGLT2) inhibitors have recently become the focus of substantial research. Some SGLT2 inhibitors improved cardiovascular outcomes in patients with type 2 diabetes [4, 5]. The Dapagliflozin Effect on CardiovascuLAR Events [DECLARE-TIMI 58] trial showed the favorable effect of dapagliflozin, one of the SGLT2 inhibitors, compared to the effects of placebo on suppression of hospitalization for heart failure with neutral effect on the following major adverse cardiovascular events: cardiovascular death, non-fatal myocardial infarction, or non-fatal ischemic stroke (MACE) [6]. On the other hand, the results of placebo-controlled non-inferiority randomized clinical trials suggested that DPP-4 inhibitors have a neutral effect of cardiovascular events in patients with type 2 diabetes [7–9].

Some studies have compared SGLT2 inhibitors and DPP-4 inhibitors regarding their glucose-lowering effect [10–14], and recent comparative studies with retrospective observational design indicated that SGLT2 inhibitors were superior to DPP-4 inhibitors for preventing cardiovascular events [15, 16]. However, there is limited evidence with prospective studies on the effect of SGLT2 inhibitors versus DPP-4 inhibitors on cardiovascular risk. Strategies for the prevention of cardiovascular events in type 2 diabetes include not only improvement of HbA1c level but also avoidance of hypoglycemia [17, 18] and maintenance of optimal body weight [19]. The scope of such strategies should be considered when assessing the efficacy of SGLT2 inhibitors and DPP-4 inhibitors for glycemic control. In addition, patients included in the previous randomized controlled trial using SGLT2 inhibitors and DPP-4 inhibitors had high prevalence of cardiovascular disease with long duration of type 2 diabetes [6, 9]. Thus, the effect of such drugs on specific cardiovascular risk factors in Japanese patients with early-stage type 2 diabetes has not been clarified.

In the present study, we aimed to clarify the efficacy of dapagliflozin versus sitagliptin for improving cardiometabolic risk factors including high glycated hemoglobin (HbA1c) level, hypoglycemia, and body weight in Japanese patients with type 2 diabetes. Specifically, we assessed the success of maintaining HbA1c levels < 7.0% (53 mmol/mol) while avoiding hypoglycemia and achieving adequate body weight reduction. This prospective trial was the first study to clinically evaluate the therapeutic benefits of dapagliflozin and sitagliptin on cardiometabolic risks by focusing concomitantly on achieving target HbA1c level < 7.0% (53 mmol/mol), maintenance of sensor glucose ≥ 3.0 mmol/L or ≥ 54 mg/dL (avoidance of hypoglycemia), and ≥ 3.0% loss of body weight in patients with type 2 diabetes [20]. Notably, hypoglycemia was carefully monitored using a flash glucose monitoring (FGM) system [21]. Thus, the results of the present study may help clarify the optimal choice of oral glucose-lowering agents and establish an effective treatment strategy for preventing cardiovascular events in early-stage type 2 diabetes.

## Methods

### Study design

The DIVERSITY-CVR study is a prospective, randomized, open-label, blinded-endpoint, parallel-group, comparative study, of which the design and rationale have been reported previously [20], registered with the University Hospital Medical Information Network Clinical Trial Registry (UMIN000028014). The study protocol was approved by the ethics committee of Toho University Omori Medical Center and by the ethics review boards of all participating institutions. Written informed consent was obtained from all participants. To minimize bias, participant randomization and data collection, management, and analysis were conducted by third-party entity (Soiken Inc., Tokyo, Japan).

### Study population

The study enrolled 340 outpatients with type 2 diabetes managed at any of the 51 participating clinics in Japan. The full list of study investigators is provided in Additional file 1. Enrollment began in July 2017 and ended in June 2018. The inclusion criteria were as follows: (1) patients with type 2 diabetes who had not used any glucose-lowering agents within 8 weeks before consenting, or those who had used only metformin; (2) those with HbA1c (NGSP values) levels of ≥ 7.1% (54 mmol/mol) but not > 10.0% (86 mmol/mol); (3) those aged between 20 and 80 years; (4) those with body mass index (BMI) of ≥ 23 kg/m^2^; (5) those who could be monitored closely for medication compliance; and (6) those who provided written consent to participate in the study. The following exclusion criteria were used: (1) patients with type 1 diabetes or secondary diabetes; (2) those with a medical history of diabetic ketoacidosis; (3) those with a medical history of myocardial infarction, cerebral infarction, or stroke within 12 weeks before consenting to the study; (4) those with severe liver disease having more than fivefold higher than normal levels of AST and ALT; (5) those with renal disease [serum creatinine ≥ 1.3 mg/dL, or estimated glomerular filtration rate (eGFR) < 45 mL/min/1.73 m^2^]; (6) those with unstable hypertension or dyslipidemia within 12 weeks before consent to the study; (7) those who were pregnant or breastfeeding or were planning to become pregnant during the study; and (8) dehydrated patients [test results showed abnormalities in hematocrit or blood urea nitrogen (BUN) or patient complaints of dehydration].

### Randomization and study intervention

After obtaining informed consent, eligible subjects were randomly assigned in a 1:1 ratio to receive dapagliflozin (5.0–10 mg/day) or sitagliptin (50–100 mg/day) add-on therapy. The randomization sequence was generated using a computer-based dynamic allocation method aiming to balance key baseline characteristics (HbA1c level, BMI, and metformin dose at the time of provision of consent). After enrollment, all concomitant prescriptions were fixed. Patients who required a change in the dose of concomitant drugs or the use of additional medications such as other glucose-lowering, antihypertensive, lipid-lowering, or antiplatelet agents were excluded from the study. Baseline measurements of blood samples and FGM measurements for > 5 days were performed during the 8-week screening period. The treatment drug (dapagliflozin 5.0 mg/day or sitagliptin 50 mg/day) was administered for 24 weeks. The dose could be increased after the first 8 weeks (dapagliflozin to 10 mg/day and sitagliptin to 100 mg/day) if necessary, to achieve the target HbA1c level of < 7.0% (53 mmol/mol). The intervention start date was set as the study start date, and the assigned treatment was continued for 24 weeks.

### Study outcomes

The primary endpoint was the proportion of patients who achieved the composite endpoint of three indices from baseline to week 24: maintenance of HbA1c level ≤ 7.0% (53 mmol/mol); maintenance of sensor glucose > 3.0 mmol/L or > 54 mg/dL (avoidance of hypoglycemia); and body weight loss ≥ 3.0% relative to baseline. We chose this composite endpoint in order to minimize the bias associated with competing risk factors [22].

In addition to the individual components of the composite endpoint, secondary endpoints included the changes (relative to baseline) in the following indices: body weight and BMI; metabolic indices including systolic blood pressure, diastolic blood pressure, fasting plasma glucose, plasma insulin, and HbA1c level; lipid indices including high-density lipoprotein (HDL) and low-density lipoprotein (LDL) cholesterol; levels of serum uric acid (UA), BUN, serum creatinine, aspartate aminotransferase (AST), and alanine aminotransferase (ALT); eGFR; blood cell counts; and glucose variability indices measured using FGM. Medication adherence rate was also reported.

FGM measurements were used to estimate the number, duration, and area under the curve (AUC) of hypoglycemia episodes, defined as periods with sensor glucose ≤ 3.9 mmol/L (≤ 70 mg/dL) or < 3.0 mmol/L (< 54 mg/dL) within 24 h and from 23:00 to 06:00. The duration of hyperglycemia episodes (glucose > 10.0 mmol/L or > 180 mg/dL) was also assessed. Furthermore, the standard deviation (SD), coefficient of variation (CV), mean amplitude of glycemic excursion (MAGE), and continuous overall net glycemic action (CONGA) calculated every 2 and 6 h were evaluated. MAGE means the mean of the difference between consecutive glycemic peak and nadirs and CONGA means the SD of the glycemic differences recorded between the specific points on the FGM profile.

### Data collection schedule

Clinical and biochemical data were collected after overnight fasting at baseline and after the 24-week treatment period. Body weight was measured at the hospital or clinic, with the subjects wearing the same type of disposable examination gowns. To check for hypoglycemia, all subjects wore the Freestyle Libre Pro^®^ monitor (Abbott Diabetes Care, Tokyo, Japan) on the upper arm for 14 days during baseline screening and at week 24. At the end of the 14-day measurement period, the subjects removed the sensor by themselves and sent it to the third-party data management center, which was blinded to clinical information. The data management center downloaded the glucose data from the sensor and used dedicated software for analysis (Abbott Diabetes Care). FGM data corresponding to the first 24 h recorded were omitted from the analysis, which included data collected during the subsequent 5 days. To estimate medication adherence rates, all subjects were requested to record their daily intake of medication using a medication diary.

### Safety evaluation

During the course of the study, the patients were monitored for adverse events (AEs) through regular medical checkups. When an AE occurred, with or without any relationship with the study drug, the investigator reported the details immediately to the participating institution, principal investigator, and study administration office. All AEs, including drug-related side effects and abnormal clinical test results, were diligently reported and documented.

### Sample size calculation and statistical analysis

We used data from our previous studies [23, 24] to estimate the expected difference in the primary endpoint and determine the required sample size for this study [20]. The details of determining the sample size are described in the previous rationale report of this study [20]. Analyses for the primary and secondary endpoints were primarily performed on the full analysis set (FAS), which included all subjects assigned to a study intervention. However, subjects without data for the primary endpoint or with a significant study protocol violation were excluded from the FAS. The details of observed severe protocol violations in this study were as follows; (1) sulfonylurea was started at the same time as the dapagliflozin was administered, (2) ipragliflozin was administered, (3) insulin therapy was administered, (4) sitagliptin 25 mg was administered. Safety analysis (AE incidence) included all treated patients. Summary statistics were calculated for continuous variables. The t-test and Fisher’s exact test were used to assess differences in baseline characteristics between the groups. For the primary endpoint (proportion of participants who achieved the composite endpoint), between-group comparison was performed using the chi-square test. Additionally, for sensitivity analysis, the Mantel–Haenszel test was performed after adjusting for allocation factors (HbA1c level < 8.5%/≥ 8.5%, BMI < 27 kg/m^2^/≥ 27 kg/m^2^, metformin dose ≤ 500 mg/> 500 mg) to estimate the risk difference and its 95% confidence interval. For the secondary endpoints (change in various indices from baseline to week 24), the two-sample t-test and analysis of covariance were used; the results were expressed as adjusted mean (standard error). Analysis of covariance included HbA1c level, BMI, and dose of metformin as covariates (as described above), as well as the baseline value of each analyzed variable. For variables with non-normal distribution, data were logarithmically transformed. FGM data including the number, duration, and AUC of hypoglycemia episodes were compared between the groups using the Wilcoxon rank sum test. All statistical analyses were performed by independent staff at the administrative office of the DIVERSITY-CVR study (Soiken Inc., Tokyo, Japan), using SAS version 9.4 (SAS Institute, Cary, NC).

### Human rights and ethical principles

All investigators involved in this study complied with the World Medical Association Declaration of Helsinki (2013 revision) and Ethical Guidelines for Medical and Health Research Involving Human Subjects (December 22, 2014, Ministry of Education, Culture, Sports, Science and Technology/Ministry of Health, Labor and Welfare), as well as with other relevant bylaws and regulations.

## Results

### Clinical characteristics

**Fig. 1:**
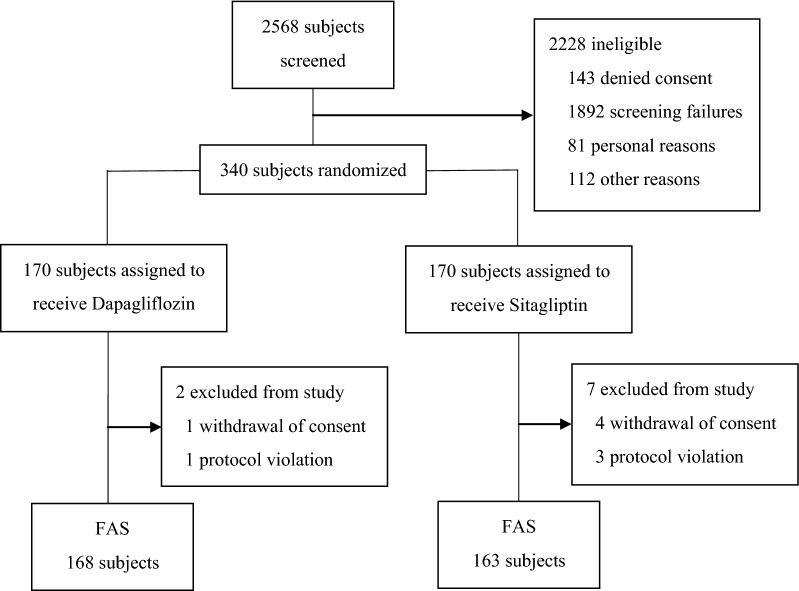
Flow chart of participant enrollment, allocation, and analysis. *FAS* full analysis set

In this study, 2568 subjects were screened and 2228 patients were ineligible (143 subjects denied consent, 1892 due to screening failure, 81 due to personal reasons, and 112 for other reasons). Three hundred forty patients were enrolled and randomized, and 331 completed the study and were included in the FAS (168 and 163 patients in the dapagliflozin and sitagliptin groups, respectively, Fig. 1). The baseline clinical characteristics are summarized in Table 1. Most patients were middle aged (average age, approximately 58 years) and overweight (average BMI, approximately 28 kg/m^2^). Disease duration was relatively short (on average, approximately 6 years), with moderate hyperglycemia (average HbA1c level, 7.8% or 62 mmol/mol). The prevalence of macrovascular complications was low (< 5.0%). Approximately 40% of patients were drug-naïve, and the average dose of biguanides was low (approximately 550 mg upon setting the dose of drug-naïve patients to 0 mg). The groups did not differ regarding any baseline clinical characteristics other than the prevalence of diabetic nephropathy, which was significantly lower in the dapagliflozin group than in the sitagliptin group (15 vs. 33, *P *< 0.05), although this difference was not reflected in the baseline eGFR (79.0 ± 18.5 vs. 78.9 ± 16.9 mL/min/1.73 m^2^). There was no between-group difference in medication adherence (97.0% vs. 97.5%).

**Table 1:** Baseline characteristics

Characteristic	Dapagliflozin group (n = 168)	Sitagliptin group (n = 163)	*P*-value
Sex (male/female) n (%)	104 (61.9)/64 (38.1)	95 (58.3)/68 (41.7)	0.57
Age (years)	58.3 ± 12.4	57.9 ± 12.1	0.71
Body weight (kg)	74.5 ± 13.4	74.9 ± 15.0	0.84
BMI (kg/m^2^)	27.8 ± 4.0	27.9 ± 4.2	0.76
Systolic blood pressure (mmHg)	134.6 ± 15.9	132.8 ± 15.7	0.28
Diastolic blood pressure (mmHg)	80.5 ± 12.1	79.1 ± 11.0	0.25
Duration of diabetes (years)	6.0 ± 6.4	5.6 ± 5.8	0.47
Fasting plasma glucose (mg/dL)	151.7 ± 33.4	152.1 ± 30.7	0.92
HbA1c (NGSP %)	7.8 ± 0.8	7.8 ± 0.8	0.90
HbA1c (mmol/mol)	61.2 ± 8.4	61.2 ± 8.3	0.90
eGFR (mL/min/1.73 m^2^)	79.0 ± 18.5	78.9 ± 16.9	0.97
Current smoking	45 (26.8)	47 (28.8)	0.71
Microvascular complications			
Diabetic retinopathy	12 (7.6)	10 (6.7)	0.83
Diabetic nephropathy	15 (9.1)	33 (20.5)	0.005
Diabetic neuropathy	12 (7.5)	7 (4.5)	0.35
Macrovascular complications			
Cerebrovascular disease	2 (1.2)	1 (0.6)	1.00
Coronary disease	7 (4.2)	4 (2.5)	0.54
Peripheral arterial disease	1 (0.6)	1 (0.6)	1.00
Anti-diabetic drugs	100 (59.5)	95 (58.3)	0.82
Biguanides	100 (59.5)	95 (58.3)	0.82
Dose of biguanides (mg)	561.9 ± 630.0	523.8 ± 577.3	0.57
Antihypertensive drugs	71 (42.3)	73 (44.8)	0.66
Diuretic drugs	10 (6.0)	20 (12.3)	0.06
Calcium channel blockers	53 (31.5)	49 (30.1)	0.81
ACE inhibitors	1 (0.6)	2 (1.2)	0.62
Angiotensin II receptor blockers	52 (31.0)	60 (36.8)	0.30
β-blockers	5 (3.0)	1 (0.6)	0.21
α-blockers	1 (0.6)	2 (1.2)	0.62
Lipid-lowering agents	72 (42.9)	71 (43.6)	0.91
Statins	60 (35.7)	56 (34.4)	0.82
Fibrates	12 (7.1)	9 (5.5)	0.65
Ezetimibe	5 (3.0)	7 (4.3)	0.57
Eicosapentaenoic acid	2 (1.2)	5 (3.1)	0.28

Data are presented as frequency (percentage) or mean ± standard deviation, as appropriate. *P*-values for between-group comparisons were obtained using the Student *t*-test and Fisher’s exact test for continuous and categorical variables, respectively

*BMI* body mass index, *HbA1c* glycated hemoglobin, *NGSP* National Glycohemoglobin Standardization Program, *eGFR* estimated glomerular filtration rate, *ACE* angiotensin converting enzyme

### Superiority of dapagliflozin regarding the primary endpoint

The proportion of patients who achieved the composite endpoint of HbA1c level maintenance ≤ 7.0% (53 mmol/mol), avoidance of hypoglycemia, and body weight loss ≥ 3.0% after 24 weeks of treatment is summarized in Table 2. We conducted analyses using the chi-square test and Mantel–Haenszel test (see also Additional file 2: Table S1). The primary endpoint (proportion of patients who achieved the composite endpoint of glucose control, hypoglycemia avoidance, and adequate body weight loss) was significantly higher in the dapagliflozin group than in the sitagliptin group (Table 2). The success of glycemic control (maintenance of HbA1c level ≤ 7.0% or 53 mmol/mol) was comparable between the groups (Table 2). Hypoglycemia (glucose < 3.0 mmol/L or < 54 mg/dL) was avoided in the majority of patients in both groups (Table 2). On the other hand, the proportion of patients who achieved ≥ 3.0% loss in body weight was significantly higher in the dapagliflozin group than in the sitagliptin group (Table 2).

**Table 2:** Achieved number or ratio regarding improvement of cardiometabolic risks in patients with early-stage type 2 diabetes

Endpoint	Dapagliflozin group (n = 160)	Sitagliptin group (n = 159)	*P*-value
	n (%)	n (%)	
Composite endpoint	39 (24.4)	22 (13.8)	0.017
HbA1c level maintenance ≤ 7.0% (53 mmol/mol)	81 (49.4)	80 (50.0)	0.91
Avoidance of hypoglycemia	141 (88.7)	144 (92.3)	0.27
More than 3.0% body weight loss	87 (54.4)	31 (19.6)	< 0.001

Data are presented as frequency (percentage). *P*-values for between-group comparisons were obtained using the chi-square test

### Effects on other cardiometabolic risk factors

Other cardiometabolic risk factors were also evaluated as secondary endpoints (Tables 3, Additional file 3: Table S2 and Additional file 4: Table S3). Although no significant differences between the groups were found regarding the change in HbA1c level, the improvement in fasting plasma glucose was significantly more pronounced in the dapagliflozin group than in the sitagliptin group: − 23.0 (2.6) vs. − 15.1 (2.6) mg/dL (*P *< 0.01; Table 3). The improvement in fasting plasma insulin was also significantly more pronounced in the dapagliflozin group than in the sitagliptin group: − 0.17 (0.06) vs. 0.17 (0.06) ln(μU/mL) (*P *< 0.001; Table 3). Regarding lipid parameters, HDL cholesterol experienced a significantly more pronounced increase in the dapagliflozin group than in the sitagliptin group: 0.07 (0.01) vs. 0.00 (0.01) mg/dL (*P *< 0.001; Table 3). However, the changes in triglyceride and LDL cholesterol were comparable between the groups. The dapagliflozin group experienced significantly more pronounced decrease in AST and ALT, increase in hematocrit, decrease in UA, and increase in BUN as well as significantly less pronounced increase in serum creatinine and decrease in eGFR (Table 3). The number, duration, and AUC of hypoglycemic episodes within 24 h and from 23:00 to 06:00 were comparable between the groups (Additional file 3: Table S2). The duration of hyperglycemia episodes was also comparable between the groups (Table 3). However, FGM data reflecting glucose variability, including SD, CV, MAGE, and CONGA (at 2 and 6 h), showed significantly larger improvement in the sitagliptin group than in the dapagliflozin group (Table 3).

**Table 3:** Summary of secondary endpoints

Variable	Adjusted mean (SE)		Adjusted mean difference (95% Cl)	*P*-value
	Dapagliflozin group (n = 168)	Sitagliptin group (n = 163)		
ΔBody weight (kg)	− 2.8 (0.3)	− 0.5 (0.3)	− 2.3 (− 2.9, − 1.7)	< 0.001
ΔBMI (kg/m^2^)	− 1.0 (0.1)	− 0.2 (0.1)	− 0.9 (− 1.1, − 0.6)	< 0.001
ΔSystolic blood pressure (mmHg)	− 3.8 (1.5)	− 1.9 (1.5)	− 1.9 (− 5.1, 1.4)	0.26
ΔDiastolic blood pressure (mmHg)	− 2.0 (1.0)	− 1.0 (1.1)	− 1.0 (− 3.3, 1.3)	0.39
ΔFasting plasma glucose (mg/dL)	− 23.0 (2.6)	− 15.1 (2.6)	− 7.9 (− 13.6, − 2.2)	0.006
ΔFasting plasma insulin (μU/mL)^a^	− 1.3 (0.9)	1.0 (0.9)	− 2.3 (− 4.1, − 0.5)	NA
ΔFasting plasma insulin [ln(μU/mL)]^a^	− 0.17 (0.06)	0.17 (0.06)	− 0.34 (− 0.46, − 0.21)	< 0.001
ΔHbA1c (NGSP%)	− 0.9 (0.1)	− 0.9 (0.1)	0.0 (− 0.1, 0.2)	0.85
ΔHbA1c (mmol/mol)	− 9.5 (0.8)	− 9.6 (0.8)	0.2 (− 1.4, 1.7)	0.85
ΔHDL cholesterol (mg/dL)^a^	4.5 (0.8)	0.2 (0.8)	4.3 (2.6, 6.0)	NA
ΔHDL cholesterol [ln(mg/dL)]^a^	0.07 (0.01)	0.00 (0.01)	0.07 (0.04, 0.10)	< 0.001
ΔLDL cholesterol (mg/dL)^a^	− 0.4 (3.0)	− 3.8 (2.9)	3.4 (− 3.4, 10.1)	NA
ΔLDL cholesterol [ln(mg/dL)]^a^	0.00 (0.02)	− 0.04 (0.02)	0.04 (− 0.01, 0.09)	0.15
ΔTriglycerides (mg/dL)^a^	− 17.6 (11.0)	− 12.2 (11.1)	− 5.5 (− 30.0, 19.1)	NA
ΔTriglycerides [ln(mg/dL)]^a^	− 0.11 (0.04)	− 0.05 (0.04)	− 0.06 (− 0.15, 0.04)	0.25
ΔAST (IU/L)^a^	− 3.6 (0.9)	1.4 (0.9)	− 5.0 (− 6.9, − 3.1)	NA
ΔAST [ln(IU/L)]^a^	− 0.12 (0.03)	0.03 (0.03)	− 0.15 (− 0.21, − 0.09)	<0.001
ΔALT (IU/L)^a^	− 7.4 (1.3)	0.5 (1.3)	− 7.9 (− 10.8, − 5.0)	NA
ΔALT [ln(IU/L)]^a^	− 0.23 (0.03)	− 0.02 (0.03)	− 0.21 (− 0.28, − 0.13)	< 0.001
ΔHematocrit (%)	2.3 (0.2)	− 0.4 (0.2)	2.7 (2.2, 3.2)	< 0.001
ΔUA (mg/dL)	− 0.5 (0.1)	0.3 (0.1)	− 0.7 (− 0.9, − 0.5)	< 0.001
ΔBUN (mg/dL)^a^	1.2 (0.4)	0.0 (0.4)	1.1 (0.3, 2.0)	NA
ΔBUN [ln(mg/dL)]^a^	0.10 (0.02)	0.02 (0.02)	0.08 (0.03, 0.13)	0.001
ΔCreatinine (mg/dL)^a^	0.0 (0.0)	0.0 (0.0)	0.0 (0.0, 0.0)	NA
ΔCreatinine [ln(mg/dL)]^a^	0.02 (0.01)	0.04 (0.01)	− 0.02 (− 0.05, 0.00)	0.025
ΔeGFR (mL/min/1.73 m^2^)	− 1.4 (0.8)	− 3.5 (0.8)	2.0 (0.2, 3.9)	0.032
ΔGlucose > 10.0 mmol/L (180 mg/dL) within 24 h				
Duration (h)	− 4.4 (0.4)	− 3.9 (0.4)	− 0.5 (− 1.5, 0.4)	0.29
ΔSD of glucose (mg/dL)	− 6.0 (0.9)	− 8.6 (0.9)	2.6 (0.7, 4.5)	0.006
ΔCV glucose (%)	1.0 (0.4)	− 1.4 (0.4)	2.4 (1.5, 3.2)	< 0.001
ΔMAGE (mg/dL)	− 14.9 (2.2)	− 22.9 (2.2)	7.9 (3.1, 12.8)	0.002
ΔCONGA for 2 h (mg/dL)	− 5.5 (0.9)	− 9.5 (0.9)	3.9 (1.9, 6.0)	< 0.001
ΔCONGA for 6 h (mg/dL)	− 9.0 (1.3)	− 13.2 (1.3)	4.2 (1.2, 7.1)	0.006

Data are presented as adjusted mean (SE) and adjusted mean difference (95% CI). *P*-values for between-group comparisons were obtained using analysis of covariance

*SE* standard error, *CI* confidence interval, *Δ* amount of change from baseline to 24 weeks, *BMI* body mass index, *HbA1c* glycated hemoglobin, *NGSP* National Glycohemoglobin Standardization Program, *HDL* high-density lipoprotein, *LDL* low-density lipoprotein, *AST* aspartate aminotransferase, *ALT* alanine aminotransferase, *UA* uric acid, *BUN* blood urea nitrogen, *eGFR* estimated glomerular filtration rate, *SD* standard deviation, *CV* coefficient of variation, *MAGE* mean amplitude of glycemic excursion, *CONGA* continuous overall net glycemic action, *NA* not applicable

^a^Variables with skewed distributions were performed log-transformed and were analyzed using log-transformed data

### Safety outcomes

During the study, 41 of 168 patients (24.3%) in the dapagliflozin group and 41 of 163 patients (24.7%) in the sitagliptin group reported AEs (Additional file 5: Table S4). No significant differences in AEs were found between the groups. No serious AEs including severe hypoglycemia or hyperglycemia were observed in either group.

## Discussion

This prospective randomized study enrolled 340 patients with early-stage type 2 diabetes (duration of diabetes: around 6 years). On average, the participants were overweight (BMI, approximately 28 kg/m^2^) and had inadequate glycemic control (HbA1c level, 7.8% or 62 mmol/mol) with metformin only or without glucose-lowering agents. The results of this study suggest that dapagliflozin is superior to sitagliptin for improving cardiometabolic risk factors in overweight Japanese patients with early-stage but inadequately controlled type 2 diabetes.

In this study, glycemic control (HbA1c level < 7.0% or 53 mmol/mol) was achieved in around 50% in both groups. Although it was reported that each 1.0% reduction in HbA1c level was significantly associated with a 14% and 12% reduction in the risk of myocardial infarction and stroke, respectively [25], intensive glycemic control can lead to more hypoglycemic episodes and does not always reduce the incidence of cardiovascular events or mortality [26]. In fact, hypoglycemia is recognized as a potent marker of high risk for cardiovascular events and mortality [18]. In the present study, we found that hypoglycemia assessed using FGM was successfully avoided in approximately 90% of participants in both groups. No significant difference was observed regarding hypoglycemia between the groups. These data indicate that both dapagliflozin and sitagliptin can be used to improve glycemic control while minimizing hypoglycemic episodes within 24 weeks of treatment in Japanese patients with type 2 diabetes.

On the other hand, ≥ 3.0% loss in body weight over the course of 24 weeks was achieved significantly more often in the dapagliflozin group (54.4% of the allocated patients). The change in body weight at 24 weeks was − 2.7 ± 3.0 kg in the dapagliflozin group and − 0.4 ± 2.6 kg in the sitagliptin group. The previous studies indicated that the change in body weight was approximately − 3.0 kg with 10 mg of dapagliflozin treatment for 24-week [6], and − 0.2 ± 0.2 kg with 100 mg of sitagliptin treatment [27]. Thus, the changes in body weight seen in this study were similar to those observed in other previous reports. Obesity is considered a major risk factor of cardiovascular disease [19]. Recently, Rosenzweig et al. reported that body weight loss of > 5.0% per year protects against cardiovascular disease in overweight individuals [28]. In overweight or obese Japanese populations, the minimum weight reduction required to improve obesity-related risk factors or conditions was reported at 3.0% [29], which was the threshold used in our present study. Recently, Reaven et al. reported significant reduction in cardiovascular risk using SGLT2 inhibitors and glucagon-like peptide 1 receptor agonists [4, 30], concluding that approaches beyond glycemic control (such as body weight reduction) represent important strategies for reducing the risk of cardiovascular events and death among populations with type 2 diabetes [31].

Although the achievement ratio of the primary composite endpoints may be regarded as low in both groups, it resulted from the multiplication of each achievement ratio of the individual components of the composite endpoint. Previous report suggested that composite endpoints were preferred to assess the clinical benefit of intervention avoiding misinterpretation associated with competing risks factor bias and challenge of using a single outcome to validate the study objective in trials on patients with diabetes [22]. Regarding the composite endpoint of cardiometabolic risk factors, our study showed the superiority of dapagliflozin compared to that of sitagliptin. It seems that the superiority of dapagliflozin stemmed mainly from the effect on body weight reduction.

In our study, dapagliflozin was more effective than sitagliptin not only regarding body weight reduction but also regarding the decrease in AST, ALT, fasting plasma glucose level, and fasting plasma insulin level. These results are consistent with those of previous reports that SGLT2 inhibitors ameliorate hepatic steatosis [32] and improve insulin sensitivity [33]. Both hepatic steatosis [34] and insulin resistance [35] are known risk factors of cardiovascular disease. Taken together, these data suggest that dapagliflozin might indeed be superior to sitagliptin for the cardiometabolic effects. In addition, previous studies reported the preferable cardiometabolic effects regarding SGLT2 inhibitors [36–39]. Dapagliflozin also showed stronger therapeutic effects on other indices that might contribute to the prevention of cardiovascular events. For example, the increase in HDL cholesterol was significantly more pronounced in the dapagliflozin group than in the sitagliptin group. Previous studies reported that an increase in HDL cholesterol concentration is associated with a decrease in the risk of coronary artery disease [40]. The increase in hematocrit count was also significantly more pronounced in the dapagliflozin group. Ferrannini et al. indicated that SGLT2 inhibitors may increase hematocrit count by stimulating erythropoiesis, which increases oxygen transport to the tissues and protects from cardiovascular events [41]. Hyperuricemia is also known as a risk factor of all-cause mortality and cardiovascular events among type 2 diabetes populations [42]. Our present finding that the decrease in UA was significantly more pronounced in the dapagliflozin group also supports the choice of dapagliflozin over sitagliptin. Recent evidence from placebo-controlled trials suggests that SGLT2 inhibitors suppress the progression of kidney disease [43] and lower the risk of kidney failure [44]. In the present study, the decrease in kidney function (assessed in terms of serum creatinine and eGFR) was significantly smaller in the dapagliflozin group than in the sitagliptin group. A previous study reported that an eGFR yearly decline of > 1.63 mL/min/1.73 m^2^ is associated with a significant increase in the incidence of major cardiovascular events [45]. These data also support the choice of dapagliflozin over sitagliptin to promote renal and cardiovascular protection in patients with early-stage type 2 diabetes.

Our present study was the first to compare the efficacy of dapagliflozin and sitagliptin in terms of glucose fluctuation evaluated using the Freestyle Libre Pro^®^ device. Interestingly, we found that sitagliptin was superior to dapagliflozin regarding glucose variability (SD, CV, MAGE, and CONGA at 2 and 6 h) assessed using FGM. Nevertheless, the change in HbA1c level was comparable between the groups and dapagliflozin provided a larger reduction in fasting plasma glucose. Taken together, these findings suggest that sitagliptin might predominantly lower postprandial blood glucose and suppress glucose fluctuation. As increased glucose variability was reportedly associated with increased risk of cardiovascular events [46, 47], sitagliptin might also contribute to the prevention of cardiovascular events through suppression of glucose variability, at least partly.

Several limitations of the study should be mentioned. First, this was an open-label study and all patients were Japanese. The Trial Evaluating Cardiovascular Outcomes with Sitagliptin reported that East Asians had the greatest HbA1c level response to sitagliptin [48]. Furthermore, the glycemic response to DPP-4 inhibitors is larger in Asian subjects than in other races [49]. Therefore, our findings that HbA1c level reduction was comparable between the groups and that the improvement in glucose variability was better for sitagliptin may not apply fully to Caucasians. Future trials with larger sample size, adequate ethnicity representation, and long-term observation are necessary to confirm the generalizability of our results. Second, the baseline prevalence of diabetic nephropathy was higher in the sitagliptin group than in the dapagliflozin group. As urine albumin level was not measured in this study, we could not evaluate the change in urine albumin levels. However, baseline eGFR was similarly well preserved in both groups (at approximately 79.0 mL/min/1.73 m^2^). Third, although we recorded AEs during the study period of 24 weeks, we did not measure surrogate markers of cardiac function or record the actual incidence of cardiovascular events. Such measurements would provide a better understanding of the preventive effects of the intervention drugs on arteriosclerosis and cardiovascular disease.

## Conclusions

To our knowledge, the DIVERSITY-CVR study was the first study to directly compare the cardiometabolic risk reduction between dapagliflozin and sitagliptin as first- or second-line therapies in Japanese patients with early-stage type 2 diabetes. Although dapagliflozin and sitagliptin provided similar effects on glycemic control with avoidance of hypoglycemic episodes, adequate loss in body weight occurred significantly more frequently in the dapagliflozin group. Additionally, various cardiometabolic indices improved to a significantly greater extent in the dapagliflozin group than in the sitagliptin group. Taken together, these data suggest that dapagliflozin therapy may be more effective for primary prevention of cardiometabolic risk factors in overweight patients with early-stage type 2 diabetes. Our findings are potentially useful in establishing an effective treatment strategy for patients with early-stage type 2 diabetes.

## Supplementary information

**Additional file 1.** Principal Investigators List.

**Additional file 2: Table S1.** Adjusted risk of endpoints that affect the incidence of cardiovascular events.

**Additional file 3: Table S2.** Hypoglycemic episodes.

**Additional file 4: Table S3.** Summary of evaluated indices.

**Additional file 5: Table S4.** Adverse events.

## Data Availability

The datasets were analyzed during the current study are available from the corresponding author on reasonable request.

## Abbreviations

AEs: adverse events
ALT: alanine aminotransferase
AST: aspartate aminotransferase
AUC: area under the curve
BMI: body mass index
BUN: blood urea nitrogen
CONGA: continuous overall net glycemic action
CV: coefficient of variation
DPP-4: dipeptidyl peptidase 4
eGFR: estimated glomerular filtration rate
FAS: full analysis set
FGM: flash glucose monitoring
HbA1c: high glycated hemoglobin
HDL: high-density lipoprotein
LDL: low-density lipoprotein cholesterol
MAGE: mean amplitude of glycemic excursion
SD: standard deviation
SGLT2: sodium-glucose cotransporter-2
UA: uric acid
