# Supplementary material for: Efficacy of dapagliflozin versus sitagliptin on cardiometabolic risk factors in Japanese patients with type 2 diabetes: a prospective, randomized study (DIVERSITY-CVR)
Source: Cardiovasc Diabetol. 2020 Jan 7;19:1. doi: 10.1186/s12933-019-0977-z (PMC6945792; doi:10.1186/s12933-019-0977-z)
Supplement: Supplementary file 1 — Additional file 1. Principal Investigators List. [file 12933_2019_977_MOESM1_ESM.doc]

**Additional file 1: Principal Investigators List.**

**List of institutions participating in the study**

Division of Diabetes, Metabolism, and Endocrinology, Department of Medicine, Toho University Graduate School of Medicine, Tokyo, Japan: Naoki Kumashiro*, Fumika Shigiyama, Takahisa Hirose, Fukumi Yoshikawa, Kayoko Ikehara, Ayako Fuchigami, Masahiko Miyagi, Hiroshi Yoshino, Genki Sato, Yuki Kojimahara, Hiroshi Uchino, Naoko Miyashita, Shigenori Hiruma, Momoko Kanaguchi, Ayana Yamamoto

Tokyo Metropolitan Cancer and Infectious Diseases Center Komagome Hospital: Toru Kitazawa*

The First Department of Internal Medicine, School of Medicine, University of Occupational and

Environmental Health: Yosuke Okada*, Maiko Hajime, Akira Kurozumi, Keiichi Torimoto

Saiseikai Yokohamashi Tobu Hospital: Mariko Higa*, Takamasa Ichijo*, Mai Hijikata

Japanese Red Cross Medical Center: Toru Hiyoshi*

Department of Endocrinology and Diabetes, School of Medicine, Saitama Medical University: Ikuo Inoue*

Kumagaya Geka Hospital: Ikuo Inoue*

Japan Community Health care Organization Tokyo Kamata Medical Center: Kaoru Iso*,

Yuka Kobayashi, Yuki Kojimahara, Satomi Konuma

Juntendo Tokyo Koto Geriatric Medical Center, Makita General Hospital: Hidenori Yoshii*

Medical Corporation Keihinkai Keihin Hospital · Shin-Keihin Hospital: Fumika Shigiyama*

Saiseikai Kanagawaken Hospital: Shuki Usui*

Department of Endocrinology and Diabetes, Nagoya University Graduate School of Medicine: Hiroshi Arima*, Takeshi Onoue

Kawasaki Rinko General Hospital: Hiroko Morioka*

Ikeda Shinryojyo: Kazuo Ikeda*

Matsuda Gastroenterology & Endocrinology clinic: Yuki Matsuda*

Shimizu Clinic: Miho Shimizu*

Daini Osaka Police Hospital: Kunihiko Hashimoto*, Ryouko Inui

Yayoi Medical Clinic: Setsuya Sakagashira*

Nippon Life Hospital: Satoru Sumitani*

Wada Clinic of Internal Medicine: Shigeo Wada*

Kotani Diabetes Clinic: Kei Kotani*

Ayame Medical Clinic: Hideo Ayame*

Nakamichi Clinic: Hitoshi Nakamichi*

Kawai Clinic: Katsuya Yamazaki*

Sato Hospital: Hiroshi Yoshino*

Isuzu Hospital: Hiromichi Koshiba*

Hasegawa Naika: Yoshiaki Hasegawa*

Sekine Clinic: Hidenori Sekine*

Iwasaki internal medicine Clinic: Shingo Iwasaki*

Kawasaki Municipal Hospital: Naoki Kumashiro*

Ikegami general hospital: Masahiko Miyagi*

Kajimoto Diabetes and endocrinology clinic: Tadafumi Kajimoto*

Sawaki Internal Medicie and Diabetes Clinic: Hideaki Sawaki*

Fuchigami Clinic: Masahiro Fuchigami*

Kumanomae Nishimura Medical Clinic: Hideki Nishimura*

Nishiki Clinic: Masateru Nishiki*

Sapporo Diabetes and Thyroid Clinic: Jun Takeuchi*

Inokuchi Clinic: Nobuo Inokuchi*

Medical Corporation Ryoshukai SennanFujii Hospital: Motoshige Miyano*

Manome Orthopadic Clinic: Naoko Miyashita*

Wakaba Eye Hospital: Fumika Shigiyama*

Kosugi Medical Clinic: Keisuke Kosugi*

Nishi Yokohama International Hospital: Masahiko Miyagi*

Johnin Ueda clinic: Kazuya Ueda*

Taniguchi Medical Clinic: Hidenori Taniguchi*

Department of Endocrinology and Diabetes Mellitus, School of Medicine, Fukuoka University: Takashi Nomiyama*, Yoshimi Muta

Matsubara Clinic: Toshiki Matsubara*

Mimihara Takasago Clinic: Hiromi Ogata*

The 3rd. Kita Shinagawa Hosp., The Khono Clin. Med. Res. Inst: Naoki Kumashiro*

Kishida clinic: Ken Kishida*

*: The representative of each institution.
