# Supplementary material for: Efficacy of dapagliflozin versus sitagliptin on cardiometabolic risk factors in Japanese patients with type 2 diabetes: a prospective, randomized study (DIVERSITY-CVR)
Source: Cardiovasc Diabetol. 2020 Jan 7;19:1. doi: 10.1186/s12933-019-0977-z (PMC6945792; doi:10.1186/s12933-019-0977-z)
Supplement: Supplementary file 2 — Additional file 2: Table S1. Adjusted risk of endpoints that affect the incidence of cardiovascular events. [file 12933_2019_977_MOESM2_ESM.doc]

**Additional file 2: Table S1. Adjusted risk of endpoints that affect the incidence of cardiovascular events**

| Endpoint | Adjusted risk (SE) | | Adjusted risk difference  (95% CI) | *P*-value |
| --- | --- | --- | --- | --- |
|  | Dapagliflozin  group  (n=160) | Sitagliptin group  (n=159) |  |  |
| Composite endpoint | 0.24 (0.03) | 0.14 (0.03) | 0.10 (0.01, 0.18) | 0.022 |
| HbA1c level maintenance ≤7.0%  (53 mmol/mol) | 0.49 (0.04) | 0.50 (0.04) | -0.01 (-0.12, 0.10) | 0.83 |
| Avoidance of hypoglycemia | 0.89 (0.02) | 0.92 (0.02) | -0.03 (-0.10, 0.03) | 0.32 |
| More than 3.0% body weight loss | 0.55 (0.04) | 0.20 (0.03) | 0.34 (0.25, 0.44) | <0.001 |

*P*-values for between-group comparisons were obtained using the Mantel-Haenszel test. SE, standard error; CI, confidence interval.
