# Supplementary material for: Efficacy of dapagliflozin versus sitagliptin on cardiometabolic risk factors in Japanese patients with type 2 diabetes: a prospective, randomized study (DIVERSITY-CVR)
Source: Cardiovasc Diabetol. 2020 Jan 7;19:1. doi: 10.1186/s12933-019-0977-z (PMC6945792; doi:10.1186/s12933-019-0977-z)
Supplement: Supplementary file 3 — Additional file 3: Table S2. Hypoglycemic episodes. [file 12933_2019_977_MOESM3_ESM.doc]

**Additional file 3: Table S2.** Hypoglycemic episodes

| Outcome | Dapagliflozin group  (n=168) | Sitagliptin group  (n=163) | *P*-value |
| --- | --- | --- | --- |
| Glucose <3.9 mmol/L (70 mg/dL) within 24 hours | |  |  |
| Events | 0.00 [0.00, 0.80] | 0.00 [0.00, 0.40] | 0.52 |
| Duration (hour) | 0.00 [0.00, 0.20] | 0.00 [0.00, 0.10] | 0.52 |
| AUC (hour×mg/dL) | 0.00 [0.00, 0.50] | 0.00 [0.00, 0.12] | 0.92 |
| Glucose <3.9 mmol/L (70 mg/dL) from 23:00 to 06:00 | |  |  |
| Events | 0.00 [0.00, 0.00] | 0.00 [0.00, 0.00] | 0.64 |
| Duration (hour) | 0.00 [0.00, 0.00] | 0.00 [0.00, 0.00] | 0.64 |
| AUC (hour×mg/dL) | 0.00 [0.00, 0.00] | 0.00 [0.00, 0.00] | 0.71 |
| Glucose <3.0 mmol/L (54 mg/dL) within 24 hours | |  |  |
| Events | 0.00 [0.00, 0.00] | 0.00 [0.00, 0.00] | 0.39 |
| Duration (hour) | 0.00 [0.00, 0.00] | 0.00 [0.00, 0.00] | 0.39 |
| AUC (hour×mg/dL) | 0.00 [0.00, 0.00] | 0.00 [0.00, 0.00] | 0.40 |
| Glucose <3.0 mmol/L (54 mg/dL) from 23:00 to 06:00 | |  |  |
| Events | 0.00 [0.00, 0.00] | 0.00 [0.00, 0.00] | 0.53 |
| Duration (hour) | 0.00 [0.00, 0.00] | 0.00 [0.00, 0.00] | 0.53 |
| AUC (hour×mg/dL) | 0.00 [0.00, 0.00] | 0.00 [0.00, 0.00] | 0.54 |

Data are presented as median [first quartile, third quartile]. *P-*values indicate results for comparisons between groups by Wilcoxon rank sum test. AUC, area under the curve.
