# Supplementary material for: Efficacy of dapagliflozin versus sitagliptin on cardiometabolic risk factors in Japanese patients with type 2 diabetes: a prospective, randomized study (DIVERSITY-CVR)
Source: Cardiovasc Diabetol. 2020 Jan 7;19:1. doi: 10.1186/s12933-019-0977-z (PMC6945792; doi:10.1186/s12933-019-0977-z)
Supplement: Supplementary file 4 — Additional file 4: Table S3. Summary of evaluated indices. [file 12933_2019_977_MOESM4_ESM.doc]

**Additional file 4: Table S3. Summary of evaluated indices**

| Variables | Dapagliflozin group  (n=168) | Sitagliptin group  (n=163) | *P*-value |
| --- | --- | --- | --- |
| Body weight (kg) |  |  |  |
| Baseline | 74.5 ± 13.4 | 74.9 ± 15.0 | 0.84 |
| Week 24 | 72.0 ± 13.6 | 74.2 ± 15.3 | 0.16 |
| Change | -2.7 ± 3.0 | -0.4 ± 2.6 | <0.001 |
| *P*-value within the group | <0.001 | 0.070 |  |
| BMI (kg/m^2^) |  |  |  |
| Baseline | 27.8 ± 4.0 | 27.9 ± 4.2 | 0.76 |
| Week 24 | 26.9 ± 4.1 | 27.8 ± 4.3 | 0.06 |
| Change | -1.0 ± 1.1 | -0.2 ± 1.0 | <0.001 |
| *P*-value within the group | <0.001 | 0.050 |  |
| Systolic blood pressure (mmHg) |  |  |  |
| Baseline | 134.6 ± 15.9 | 132.8 ± 15.7 | 0.28 |
| Week 24 | 130.4 ± 16.9 | 131.9 ± 16.3 | 0.42 |
| Change | -4.1 ± 16.3 | -1.4 ± 17.3 | 0.16 |
| *P*-value within the group | 0.002 | 0.310 |  |
| Diastolic blood pressure (mmHg) |  |  |  |
| Baseline | 80.5 ± 12.1 | 79.1 ± 11.0 | 0.25 |
| Week 24 | 78.2 ± 12.2 | 78.7 ± 11.4 | 0.73 |
| Change | -2.3 ± 11.5 | -0.4 ± 12.1 | 0.15 |
| *P*-value within the group | 0.012 | 0.680 |  |
| Fasting plasma glucose (mg/dL) |  |  |  |
| Baseline | 151.7 ± 33.4 | 152.1 ± 30.7 | 0.92 |
| Week 24 | 130.8 ± 22.9 | 139.6 ± 31.5 | 0.005 |
| Change | -19.1 ± 30.1 | -12.9 ± 32.3 | 0.09 |
| *P*-value within the group | <0.001 | <0.001 |  |
| Fasting plasma insulin (µU/mL)* |  |  |  |
| Baseline | 10.8 ± 7.1 | 12.1 ± 9.8 | NA |
| Week 24 | 9.2 ± 8.9 | 12.2 ± 7.5 | NA |
| Change | -1.5 ± 8.2 | 0.3 ± 7.9 | NA |
| *P*-value within the group | NA | NA |  |
| Fasting plasma insulin [ln(µU/mL)]* |  |  |  |
| Baseline | 2.18 ± 0.67 | 2.25 ± 0.69 | 0.38 |
| Week 24 | 1.95 ± 0.71 | 2.32 ± 0.61 | <0.001 |
| Change | -0.23 ± 0.55 | 0.09 ± 0.47 | <0.001 |
| *P*-value within the group | <0.001 | 0.046 |  |
| HbA1c (NGSP%) |  |  |  |
| Baseline | 7.8 ± 0.8 | 7.8 ± 0.8 | 0.90 |
| Week 24 | 7.1 ± 0.7 | 7.1 ± 0.8 | 0.92 |
| Change | -0.7 ± 0.8 | -0.7 ± 0.8 | 0.72 |
| *P*-value within group | <0.001 | <0.001 |  |
| HbA1c (mmol/mol) |  |  |  |
| Baseline | 61.2 ± 8.4 | 61.4 ± 8.3 | 0.90 |
| Week 24 | 53.7 ± 7.3 | 53.8 ± 9.2 | 0.92 |
| Change | -7.2 ± 8.7 | -7.6 ± 9.0 | 0.72 |
| *P*-value within group | <0.001 | <0.001 |  |
| HDL cholesterol (mg/dL)* |  |  |  |
| Baseline | 53.4 ± 13.7 | 53.5 ± 13.6 | NA |
| Week 24 | 56.5 ± 16.0 | 52.9 ± 13.1 | NA |
| Change | 3.6 ± 7.9 | -0.8 ± 7.7 | NA |
| *P*-value within group | NA | NA |  |
| HDL cholesterol [ln(mg/dL)]* |  |  |  |
| Baseline | 3.95 ± 0.25 | 3.95 ± 0.23 | 0.83 |
| Week 24 | 4.00 ± 0.26 | 3.94 ± 0.23 | 0.036 |
| Change | 0.06 ± 0.13 | -0.01 ± 0.13 | <0.001 |
| *P*-value within the group | <0.001 | 0.19 |  |
| LDL cholesterol (mg/dL)* |  |  |  |
| Baseline | 109.7 ± 34.0 | 110.5 ± 27.3 | NA |
| Week 24 | 112.6 ± 38.5 | 108.1 ± 31.2 | NA |
| Change | 2.5 ± 33.2 | -1.1 ± 22.3 | NA |
| *P*-value within the group | NA | NA |  |
| LDL cholesterol [ln(mg/dL)]* |  |  |  |
| Baseline | 4.65 ± 0.31 | 4.67 ± 0.26 | 0.54 |
| Week 24 | 4.68 ± 0.30 | 4.64 ± 0.30 | 0.32 |
| Change | 0.02 ± 0.22 | -0.02 ± 0.20 | 0.12 |
| *P*-value within the group | 0.27 | 0.27 |  |
| Triglycerides (mg/dL)* |  |  |  |
| Baseline | 159.7 ± 112.4 | 164.9 ± 150.5 | NA |
| Week 24 | 147.8 ± 121.3 | 157.9 ± 134.7 | NA |
| Change | -11.3 ± 120.5 | -9.5 ± 123.2 | NA |
| *P*-value within the group | NA | NA |  |
| Triglycerides [ln(mg/dL)]* |  |  |  |
| Baseline | 4.91 ± 0.55 | 4.92 ± 0.54 | 0.81 |
| Week 24 | 4.80 ± 0.57 | 4.87 ± 0.56 | 0.27 |
| Change | -0.10 ± 0.48 | -0.05 ± 0.40 | 0.37 |
| *P*-value within the group | 0.012 | 0.11 |  |
| AST (IU/L)* |  |  |  |
| Baseline | 25.2 ± 10.5 | 27.6 ± 14.2 | NA |
| Week 24 | 22.4 ± 8.0 | 29.2 ± 15.6 | NA |
| Change | -2.8 ± 7.2 | 1.5 ± 11.1 | NA |
| *P*-value within the group | NA | NA |  |
| AST [ln(IU/L)]* |  |  |  |
| Baseline | 3.15 ± 0.37 | 3.22 ± 0.43 | 0.16 |
| Week 24 | 3.06 ± 0.31 | 3.26 ± 0.47 | <0.001 |
| Change | -0.10 ± 0.25 | 0.04 ± 0.32 | <0.001 |
| *P*-value within the group | <0.001 | 0.15 |  |
| ALT (IU/L)* |  |  |  |
| Baseline | 31.5 ± 18.6 | 34.5 ± 23.7 | NA |
| Week 24 | 26.1 ± 15.7 | 36.1 ± 25.8 | NA |
| Change | -5.7 ± 11.9 | 1.6 ± 15.9 | NA |
| *P*-value within the group | NA | NA |  |
| ALT [ln(IU/L)]* |  |  |  |
| Baseline | 3.29 ± 0.58 | 3.36 ± 0.58 | 0.25 |
| Week 24 | 3.11 ± 0.54 | 3.38 ± 0.63 | <0.001 |
| Change | -0.18 ± 0.34 | 0.02 ± 0.37 | <0.001 |
| *P*-value within the group | <0.001 | 0.58 |  |
| Hematocrit (%) |  |  |  |
| Baseline | 43.1 ± 3.6 | 43.0 ± 3.9 | 0.81 |
| Week 24 | 45.2 ± 4.2 | 42.4 ± 3.9 | <0.001 |
| Change | 2.2 ± 2.3 | -0.5 ± 2.0 | <0.001 |
| *P*-value within the group | <0.001 | 0.004 |  |
| UA (mg/dL) |  |  |  |
| Baseline | 5.4 ± 1.3 | 5.4 ± 1.4 | 0.81 |
| Week 24 | 4.9 ± 1.1 | 5.6 ± 1.3 | <0.001 |
| Change | -0.5 ± 0.9 | 0.2 ± 0.9 | <0.001 |
| *P*-value within the group | <0.001 | 0.004 |  |
| BUN (mg/dL)* |  |  |  |
| Baseline | 14.2 ± 4.2 | 14.7 ± 7.3 | NA |
| Week 24 | 15.5 ± 4.1 | 14.6 ± 4.4 | NA |
| Change | 1.4 ± 3.7 | -0.2 ± 7.1 | NA |
| *P*-value within the group | NA | NA |  |
| BUN [ln(mg/dL)]* |  |  |  |
| Baseline | 2.61 ± 0.29 | 2.62 ± 0.33 | 0.70 |
| Week 24 | 2.71 ± 0.26 | 2.64 ± 0.28 | 0.022 |
| Change | 0.10 ± 0.23 | 0.01 ± 0.28 | 0.003 |
| *P*-value within the group | <0.001 | 0.59 |  |
| Creatinine (mg/dL)* |  |  |  |
| Baseline | 0.8 ± 0.2 | 0.7 ± 0.2 | NA |
| Week 24 | 0.8 ± 0.2 | 0.8 ± 0.2 | NA |
| Change | 0.0 ± 0.1 | 0.0 ± 0.1 | NA |
| *P*-value within the group | NA | NA |  |
| Creatinine [ln(mg/dL)]* |  |  |  |
| Baseline | -0.31 ± 0.24 | -0.33 ± 0.23 | 0.53 |
| Week 24 | -0.29 ± 0.24 | -0.29 ± 0.24 | 0.75 |
| Change | 0.02 ± 0.10 | 0.04 ± 0.10 | 0.022 |
| *P*-value within the group | 0.019 | <0.001 |  |
| eGFR (mL/min/1.73 m^2^) |  |  |  |
| Baseline | 79.0 ± 18.5 | 78.9 ± 16.9 | 0.97 |
| Week 24 | 77.0 ± 18.5 | 75.3 ± 17.1 | 0.40 |
| Change | -1.6 ± 8.7 | -3.6 ± 8.2 | 0.041 |
| *P*-value within the group | 0.027 | <0.001 |  |
| SD of glucose (mg/dL) | |  |  |
| Baseline | 43.5 ± 10.7 | 43.0 ± 10.6 | 0.70 |
| Week 24 | 37.5 ± 9.6 | 34.6 ± 10.5 | 0.010 |
| Change | -5.9 ± 10.1 | -8.2 ± 9.8 | 0.036 |
| *P*-value within group | <0.001 | <0.001 |  |
| CV glucose (%) |  |  |  |
| Baseline | 25.6 ± 4.8 | 25.0 ± 4.5 | 0.31 |
| Week 24 | 26.2 ± 4.7 | 23.4 ± 4.7 | <0.001 |
| Change | 0.5 ± 4.5 | -1.6 ± 4.0 | <0.001 |
| *P*-value within the group | 0.16 | <0.001 |  |
| MAGE (mg/dL) |  |  |  |
| Baseline | 110.8 ± 25.9 | 109.3 ± 28.8 | 0.62 |
| Week 24 | 95.2 ± 23.8 | 86.5 ± 26.7 | 0.002 |
| Change | -15.2 ± 25.2 | -22.3 ± 27.7 | 0.019 |
| *P*-value within the group | <0.001 | <0.001 |  |
| CONGA (mg/dL) |  |  |  |
| 2 hours |  |  |  |
| Baseline | 52.9 ± 11.2 | 52.7 ± 12.2 | 0.88 |
| Week 24 | 47.0 ± 10.6 | 42.9 ± 11.5 | <0.001 |
| Change | -5.8 ± 10.3 | -9.5 ± 10.7 | <0.001 |
| *P*-value within the group | <0.001 | <0.001 |  |
| 6 hours |  |  |  |
| Baseline | 63.8 ± 17.9 | 62.7 ± 16.4 | 0.57 |
| Week 24 | 54.5 ± 15.6 | 49.8 ± 16.0 | 0.008 |
| Change | -9.1 ± 16.8 | -12.7 ± 15.2 | 0.049 |
| *P*-value within the group | <0.001 | <0.001 |  |

Data are presented as mean ± standard deviation. *P-*values indicate results for comparisons between groups by *t* test. *Variables with skewed distributions were performed log-transformed and were analyzed using log-transformed data. BMI, body mass index; HbA1c, glycated hemoglobin; NGSP, National Glycohemoglobin Standardization Program; HDL, high-density lipoprotein; LDL, low-density lipoprotein; AST, aspartate aminotransferase; ALT, alanine aminotransferase; UA, uric acid; BUN, blood urea nitrogen; eGFR, estimated glomerular filtration rate; SD, standard deviation; CV, coefficient of variation; MAGE, mean amplitude of glycemic excursion; CONGA, continuous overall net glycemic action; NA, not applicable.
