# Supplementary material for: Efficacy of dapagliflozin versus sitagliptin on cardiometabolic risk factors in Japanese patients with type 2 diabetes: a prospective, randomized study (DIVERSITY-CVR)
Source: Cardiovasc Diabetol. 2020 Jan 7;19:1. doi: 10.1186/s12933-019-0977-z (PMC6945792; doi:10.1186/s12933-019-0977-z)
Supplement: Supplementary file 5 — Additional file 5: Table S4. Adverse events. [file 12933_2019_977_MOESM5_ESM.doc]

**Additional file 5: Table S4. Adverse events**

| Event | Dapagliflozin group  (n=169) | Sitagliptin group  (n=166) | *P*-value |
| --- | --- | --- | --- |
|  | n (%) | n (%) |  |
| Any adverse events | 41 (24.3) | 41 (24.7) | 1.00 |
| Allergic rhinitis | 2 (1.2) | 1 (0.6) | 1.00 |
| Anemia | 1 (0.6) | 0 (0.0) | 1.00 |
| Anisakiasis | 1 (0.6) | 0 (0.0) | 1.00 |
| Anorexia | 1 (0.6) | 0 (0.0) | 1.00 |
| Arthritis | 0 (0.0) | 1 (0.6) | 0.50 |
| Atrial fibrillation | 0 (0.0) | 1 (0.6) | 0.50 |
| Atrial flutter | 1 (0.6) | 0 (0.0) | 1.00 |
| Back pain | 0 (0.0) | 2 (1.2) | 0.24 |
| Cervical polyp | 1 (0.6) | 0 (0.0) | 1.00 |
| Colon cancer | 1 (0.6) | 0 (0.0) | 1.00 |
| Constipation | 3 (1.8) | 1 (0.6) | 0.62 |
| Cystitis | 2 (1.2) | 0 (0.0) | 0.50 |
| Dehydration | 1 (0.6) | 0 (0.0) | 1.00 |
| Diarrhea | 3 (1.8) | 1 (0.6) | 0.62 |
| Dizzy | 0 (0.0) | 1 (0.6) | 0.50 |
| Eosinophilic sinusitis | 1 (0.6) | 0 (0.0) | 1.00 |
| Exanthema | 1 (0.6) | 1 (0.6) | 1.00 |
| Eyelid tumor | 1 (0.6) | 0 (0.0) | 1.00 |
| Facial nerve disorder | 0 (0.0) | 1 (0.6) | 0.50 |
| Fracture | 1 (0.6) | 0 (0.0) | 1.00 |
| Frequent urination | 1 (0.6) | 0 (0.0) | 1.00 |
| Hepatic dysfunction | 0 (0.0) | 2 (1.2) | 0.24 |
| Hypertension | 2 (1.2) | 5 (3.0) | 0.28 |
| Hypoglycemia | 1 (0.6) | 0 (0.0) | 1.00 |
| Hypotension | 1 (0.6) | 0 (0.0) | 1.00 |
| Hypothyroidism | 0 (0.0) | 1 (0.6) | 0.50 |
| Ingrown toenail | 1 (0.6) | 0 (0.0) | 1.00 |
| Insomnia | 0 (0.0) | 1 (0.6) | 0.50 |
| Limb pain | 0 (0.0) | 1 (0.6) | 0.50 |
| Nausea | 1 (0.6) | 0 (0.0) | 1.00 |
| Osteoarthritis | 0 (0.0) | 1 (0.6) | 0.50 |
| Pharyngitis | 0 (0.0) | 1 (0.6) | 0.50 |
| Pneumonia | 2 (1.2) | 0 (0.0) | 0.50 |
| Rheumatoid arthritis | 1 (0.6) | 0 (0.0) | 1.00 |
| Sciatic neuralgia | 1 (0.6) | 1 (0.6) | 1.00 |
| Spinal stenosis | 0 (0.0) | 1 (0.6) | 0.50 |
| Subacute thyroiditis | 1 (0.6) | 0 (0.0) | 1.00 |
| Swelling of thyroid nodule | 0 (0.0) | 1 (0.6) | 0.50 |
| Tinea | 1 (0.6) | 0 (0.0) | 1.00 |
| Trichomoniasis | 1 (0.6) | 0 (0.0) | 1.00 |
| Upper respiratory inflammation | 3 (1.8) | 3 (1.8) | 1.00 |
| Ureterolith | 0 (0.0) | 1 (0.6) | 0.50 |
| Urethritis | 1 (0.6) | 0 (0.0) | 1.00 |
| Urge incontinence | 0 (0.0) | 1 (0.6) | 0.50 |
| Urolithiasis | 0 (0.0) | 1 (0.6) | 0.50 |
| Uterine fibroids | 1 (0.6) | 0 (0.0) | 1.00 |
| Vaginitis | 1 (0.6) | 0 (0.0) | 0.50 |
| Weight loss | 1 (0.6) | 0 (0.0) | 1.00 |

Data are presented as frequency (percentage). *P*-values for between-group comparisons were

obtained using Fisher's exact test.
